# Editorial: From genes to grains: advancements in understanding seed development and grain filling

**DOI:** 10.3389/fpls.2025.1701948

**Published:** 2025-10-07

**Authors:** Shi-Kai Cao, Nenghui Ye, Zhenning Teng, Birendra Prasad Shaw, Rui Liu, Jianhua Zhang

**Affiliations:** ^1^ College of Life Science, Yangtze University, Jingzhou, China; ^2^ College of Agronomy, Hunan Agricultural University, Changsha, China; ^3^ Yuelushan Laboratory, Changsha, China; ^4^ Institute of Life Sciences, Bhubaneswar, India; ^5^ Department of Biology, Hong Kong Baptist University, Hong Kong, Hong Kong SAR, China; ^6^ State Key Laboratory of Agrobiotechnology, The Chinese University of Hong Kong, Hong Kong, Hong Kong SAR, China

**Keywords:** crops, seed development, grain filling, genetic regulation, environmental factors, metabolic pathways

Crop seeds serve as fundamental dietary components for humans, essential feed for livestock, and key industrial raw materials (Troyer, 2006). Seed development and grain filling are complex biological processes that govern critical agronomic traits. These highly complex processes govern critical agronomic traits through dynamic interactions involving polygenic networks, multifactorial pathways, and environmental cues, ultimately defining crop yield and grain quality. Deciphering these intricate regulatory mechanisms is therefore crucial for enhancing agricultural productivity.

This Research Topic brings together five innovative studies that employ advanced genetic, molecular, and physiological strategies to optimize crop productivity and stress adaptation. The contributions are organized into three thematic areas to effectively highlight key findings: 1) from genetics to physiology, 2) mechanistic insights into grain development, and 3) broadening to environmental adaptation.

## From genetics to physiology

As a cornerstone of modern agriculture, Heterosis (hybrid vigor) occurs when hybrid offspring exceed parental performance levels, driving revolutionary advances in crop breeding (Huang et al., 2016). Although widely observed, the genetic architecture underlying heterosis in rice grain yield remains incompletely elucidated. Addressing this gap, Ouyang et al. demonstrated that strategic combination of two major functional alleles of the *NARROW LEAF1* (*NAL1*) gene leads to hybrid plants with an optimal, mid-parental expression level of NAL1 protein. This genetic configuration conferred significant physiological benefits, including markedly increased grain yield, higher panicle number, improved plant architecture, and superior canopy photosynthetic efficiency compared to homozygous parents. By delineating the molecular basis of NAL1-mediated heterosis, this study establishes a direct pathway from allele-specific manipulation to measurable physiological gains, offering a viable strategy for substantially increasing yield in hybrid rice.

## Mechanistic insights into grain development

Recent research has provided complementary insights into grain development, spanning phytohormonal to epigenetic regulation. Grain filling in rice is tightly controlled by sophisticated phytohormonal networks that spatially and temporally regulate photoassimilate allocation, creating distinct metabolic patterns between superior and inferior grains (Teng et al., 2021; Ma et al., 2023). In a landmark study, Chandran et al. showed that both abscisic acid (ABA) and combined ABA-benzyladenine (BA) applications significantly improve yield by increasing grain weight across all spikelet positions. Their analysis revealed two distinct mechanisms: ABA primarily enhances source-to-sink photosynthate translocation, while ABA+BA induces synergistic transcriptional reprogramming of cell cycle and endoreduplication processes, specifically addressing inferior spikelet development. Simultaneously, advances in understanding the triploid endosperm—a key nutrient storage tissue coordinating seed development—have uncovered complex regulatory networks (Dai et al., 2021). Wei et al. comprehensively reviewed the role of non-coding RNAs (ncRNAs) in endosperm development, showing how ncRNAs integrate epigenetic, transcriptional, and post-transcriptional regulation to control nutrient accumulation and storage compound synthesis. These studies collectively provide a multi-scale perspective on grain development, offering complementary frameworks for next-generation crop improvement.

## Environmental adaptation

Heavy metal stress poses a major environmental challenge to agriculture, disrupting physiological processes and causing significant yield loss (Hu and Wiatrak, 2012). In an important advance, Li et al. identified a novel rapeseed (*Brassica napus*) germplasm with exceptional heavy metal tolerance and phytoextraction capacity. Integrated transcriptomic and proteomic analyses revealed conserved molecular mechanisms—including key components of oxidative stress response and metal transport—underpinning plant adaptation to contaminated environments. This work provides both a valuable genetic resource for breeding resilient crops and a dual-benefit strategy combining soil remediation with sustainable oilseed production. Complementing this, Ning et al. used ¹³C isotopic tracing to demonstrate how genetic variation among early-season rice cultivars shapes distinct carbon-nitrogen allocation strategies under varying nitrogen regimes. Their study showed that dynamic regulation of nitrogen trafficking fine-tunes source-sink photoassimilate partitioning, optimizing yield components under fluctuating nutrient conditions. This research illuminates how plants integrate environmental signals with physiological processes, providing a framework for climate-resilient cropping strategies through improved nutrient management and cultivar selection.

## Concluding perspective

This Research Topic deciphers the interconnected biological hierarchies governing crop productivity—from genetic variation and molecular regulation to whole-plant physiology and environmental adaptation. The mechanistic insights into heterosis, grain development, and stress resilience presented here establish a multidisciplinary framework for addressing pressing agricultural challenges. Together, these contributions illustrate how integrating allele-specific breeding, hormonal engineering, epigenetic modulation, and precision nutrient management represents a new paradigm in precision agriculture. Beyond deepening fundamental understanding of plant biology, this Research Topic provides tangible strategies for developing climate-resilient crops, enhancing resource-use efficiency, and advancing sustainable food security objectives in a changing global environment.
